# Gellan Gum and Polyvinyl Alcohol Based Triple-Layer Films Enriched with *Alhagi sparsifolia* Flower Extract: Preparation, Characterization, and Application of Dried Shrimp Preservation

**DOI:** 10.3390/foods12213979

**Published:** 2023-10-31

**Authors:** Yijing Yue, Xiaoyu Cheng, Haijie Liu, Mingwu Zang, Bing Zhao, Xin Zhao, Le Wang

**Affiliations:** 1 College of Food Science and Nutrition Engineering, China Agricultural University, 17 Qinghua East Road, Haidian District, Beijing 100083, China; yueyijing0213@163.com; 2 China Meat Research Center, 70 Yangqiao, Fengtai District, Beijing 100068, China; zangmw@126.com (M.Z.); zhaobtg@163.com (B.Z.); 13621073754@163.com (X.Z.); wlicecream@163.com (L.W.)

**Keywords:** anti-oxidation, dried shrimps, packaging film, preservation

## Abstract

To meet the demand for biobased packaging and minimize the oxidation of dried aquatic goods during storage, we created a triple-layer film (TF) with antioxidant capacity. The film was produced using polyvinyl alcohol (PVA) as the protective layer, gellan gum (GG)/PVA composite incorporating *Alhagi sparsifolia* flower extract (AFE) as the anti-oxidative capability layer, and GG as the anti-oxidative capacity slow-release control layer. The TFs with different AFE additions were characterized and compared to a single-layer film (SF) made of the same material. The results demonstrate that adding AFE to films degraded their water vapour and oxygen barrier properties as well as their tensile strength, but increased their light barrier properties, elongation at break, and anti-oxidative capability. The three-layer structure increased the light, water vapour, and oxygen barrier qualities of films, as well as their slow-release anti-oxidative capability. The application experiment revealed that the inclusion of AFE might aid in the preservation of dried prawn quality. Using TF supplemented with 5 (*w*/*v*) AFE to package the dried shrimps reduced the TBARS value by 47.5%. Our research indicated that TFs containing AFE have a wide range of possible applications in dried shrimp preservation.

## 1. Introduction

Biobased packaging films composed of macromolecular biopolymers such as polysaccharides and proteins are gaining popularity. Polysaccharide compounds such as starch, chitosan, carrageenan, and others have been intensively explored as film substrates due to their good moulding, cheap cost, and non-toxic properties [1]. Instead of petroleum-based plastic, biobased materials not only serve to alleviate the ecological environmental pollution produced by plastic but also help to minimize economic expenses [2]. To improve the application potential of bio-based films in food packaging, some active substances with antioxidant, antibacterial, or food quality indicating properties are incorporated into them, greatly increasing the functionality of biobased packaging films [3].

Gellan gum (GG) and PVA are both biobased materials. GG is an anionic linear heteropolysaccharide that is produced through the aerobic fermentation of the bacteria Sphingomonas elodea [4]. The substance exhibits non-toxicity, harmlessness, biodegradability, affordability, and favourable characteristics in terms of film formation [5]. GG is composed of tetrasaccharide repeat units, namely two units of D-glucose, one unit of D-glucuronic acid, and one unit of L-rhamnose [6]. Polyvinyl alcohol (PVA) is a biopolymer with polyhydroxyl qualities that does not depend on petroleum. It possesses several advantageous characteristics, including stable physical and chemical properties, non-toxicity, and harmlessness. As a result, PVA finds extensive application in the domains of wound dressing and drug delivery [7]. In numerous contemporary research investigations, PVA is included in polysaccharide-based films with the aim of enhancing their mechanical properties [8].

Dried seafood exhibits a significantly extended shelf life in comparison to alternative seafood items. Nevertheless, throughout the preservation procedure, the dried seafood will continue to experience gradual protein oxidation and lipid oxidation, significantly compromising its palatability and nutritional value [9]. Numerous bio-based packaging films possessing anti-oxidative characteristics have been created and utilized for the purpose of food preservation. *Alhagi sparsifolia* is a perennial herbaceous plant or subshrub mostly distributed in the regions of Gansu, Inner Mongolia, Xinjiang Uygur Autonomous Region, and certain areas of Central Asia. The medicinal properties of *A. sparsifolia* encompass the utilization of its sugar, flowers, and seeds. The aqueous extract of the *A. sparsifolia* flower exhibits notable anti-inflammatory and anti-oxidative properties [10], whereas the alcoholic extract of *A. sparsifolia* flower demonstrates antibacterial and hepatoprotective effects [11]. The *A. sparsifolia* flower has been determined to possess a high concentration of catechins, anthocyanins, flavonoids, and other bioactive antioxidants [12]. So far, no research has been conducted about the application of *A. sparsifolia* flower extract (AFE) in the context of food. The application in our research not only imparts a distinctive anti-oxidative effect on the film, but also facilitates the exploration and utilization of *A. sparsifolia*. Despite the numerous advantages associated with the utilization of biobased packaging film coated with active compounds, it is important to acknowledge that this approach still faces limitations in terms of its barrier and mechanical qualities. Numerous research endeavours have been undertaken to enhance the efficiency of bio-based packaging films, resulting in the development of diverse variants of multilayer films. When comparing single-layer film to multi-layer film, it is evident that the latter exhibits superior mechanical and barrier qualities, as well as the ability to release active chemicals at a slower rate [13].

To the best of our knowledge, there is a lack of study on the utilization of AFE, in the context of packaging film. Furthermore, there is a dearth of novel studies exploring the development of triple-layer films (TFs) employing GG and PVA as the substrate. This study aimed to develop a biobased packaging film with anti-oxidative properties by incorporating AFE into mixes of GG and PVA, resulting in a triple-layer structure. The efficacy and practicality of single-layer films (SFs) and TFs with varying amounts of AFE added were assessed and implemented for the purpose of preserving dried shrimp. This study offers a novel viewpoint on the multifaceted and practical advancement and utilization of biobased packaging materials.

## 2. Materials and Methods

### 2.1. Materials

Gellan Gum (low acyl, 99%) was supplied by Zhejiang Top Ingredients Co., Ltd. (XingPing, China); polyvinyl alcohol, glycerin, acetic acid, and ethanol were obtained from Sinopharm Chemical Regent Co., Ltd. (Beijing, China). The dried *A*. *sparsifolia* flowers were obtained from the local farmers (Xinjiang, China). Frozen (−20 °C) *Litopenaeus vannamei* shrimps from the same batch were commercially purchased from Beijing Beishui Food Industry Co., Ltd. (Beijing, China). Protein Content Assay Kit (Biuret Method) and Total Antioxidant Capacity (T-AOC) Assay Kit (FRAP Method) were purchased from Solarbio Science & Technology Co., Ltd. (Beijing, China).

### 2.2. Extraction of A. sparsifolia Flowers

The extract of *A. sparsifolia* flower was prepared according to Neamah [10] with modification. The desiccated *A. sparsifolia* flower were pulverized into a fine powder utilizing a high-velocity blender (JSP-100, Jinsui machinery factory, Guangzhou, China). Flower powder (500 g) was combined with purified water (2 L) and subjected to boiling for 2 h. Following the boiling process, the brown extracting solution underwent filtration under decreased pressure. Subsequently, the filtrated extraction solution was gathered and subjected to lyophilization. The powdered extraction was stored in a sealed environment and maintained at a temperature of 4 °C until the subsequent utilization.

### 2.3. Preparation of SFs and TFs

**Figure 1:**
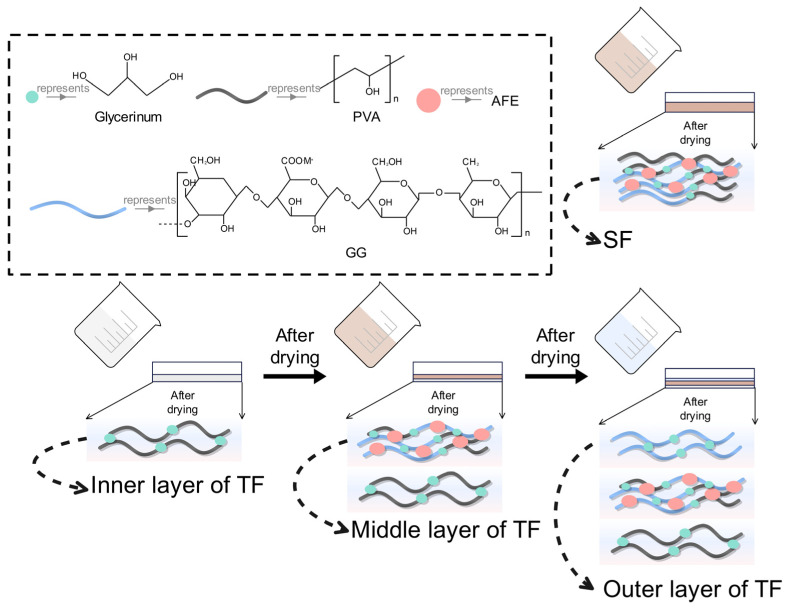
Methods for preparing SF and TF. Abbreviations: GG—gellan gum, PVA—polyvinyl alcohol, AFE—*Alhagi sparsifolia* flower extract, SF—single-layer film, TF—triple-layer film.

SFs and TFs were made utilizing the one-step and three-step solution casting methods (Figure 1), as previously described [14]. After stirring PVA (20.00 g) and GG (10.00 g) in filtered water (1 L) for 4 h at 100 °C, a SF solution was formed. Subsequently, solutions containing 1% PVA (*w*/*v*), 1% PVA + 0.5% GG (*w*/*v*), and 0.5% GG (*w*/*v*) were created using the same method as the SF solution, respectively. These solutions were used to form the outer, middle, and inner layers of the TF. Then, AFE powders with concentrations of 1.25%, 2.5%, 3.75%, and 5% (*w*/*v*) were added into the SF solution and the middle-layer solution. When the addition of AFE was more than 0.5% (*w*/*v*), the SF solution easily formed a gel, which would make it impossible to uniformly pour into the petri dish. Subsequently, 20% glycerinum (*w*/*w*) as a plasticizer was added into each film solution with continuous stirring for 10min at 25 °C.

SF solution (90 mL) was carefully poured into a petri dish (250 mm × 250 mm × 18 mm). The dish was dried for 8 h at 55 °C and 30% relative humidity to form a SF. Regarding TF, the outer layer solution (90 mL) was placed into a petri dish (250 mm × 250 mm × 18 mm) and dried using the same process as the SF. The middle-layer solution was carefully poured on the outer layer and dried under identical conditions. Finally, the inner-layer solution (90 mL) was poured on the middle layer and dried under similar conditions, resulting in the formation of TF.

### 2.4. Film Characterization

#### 2.4.1. Colour Measurements

The colour measurement was conducted using a chroma meter (CM-700d, Konica Minolta Holdings, Inc., Tokyo, Japan) against the background of a white standard plate. The L* (lightness), a* (redness), and b* (yellowness) values were measured for each sample at six randomly selected places. Total colour difference (*W*) from white standard was calculated using the following formula [14]:

(1) $$W=\sqrt{{\left(L^{*}-L_{0}^{*}\right)}^{2}+{\left(a^{*}-a_{0}^{*}\right)}^{2}+{\left(b^{*}-b_{0}^{*}\right)}^{2}}$$

L*, a*, and b* were the colour parameters of films, $L_{0}^{*}$ = 99.44 ± 0.01, $a_{0}^{*}$ = −0.07 ± 0.01, and $b_{0}^{*}$ = −0.16 ± 0.01 were the colour parameters of the standard white.

#### 2.4.2. Scanning Electron Microscopy (SEM)

The surface and cross-section of films were observed using a scanning electron microscope (S4800, Hitachi Co., Ltd., Tokyo, Japan). The films’ cross-sections were acquired through the process of fracturing in a liquid nitrogen environment. The films’ surface image was captured from the side of the preparation that was in contact with the air. Before conducting the observation, the samples underwent a process of sputter-coating with a thin layer of gold using an ion sputter coater (MC1000, Hitachi Co., Ltd., Tokyo, Japan).

#### 2.4.3. Fourier Transform Infrared Spectroscopy (FTIR) Analysis

FTIR analysis was performed using a FTIR spectrometer (Nicolet iS10, Thermo Fisher Scientific Inc., Waltham, MA, USA). The scanning rate was 4 cm^−1^ and the number of cumulative scans was 64.

#### 2.4.4. Mechanical Properties

Tensile tests of films were carried out using an auto-tensile tester (XLW-EC, Labthink Instruments Co., Ltd., Jinan, China). The films were cut into strips (150 × 15 mm). The initial distance was 100 mm and the test speed was 100 mm/min. Tensile strength (TS, MPa) and elongation at break (EAB, %) were calculated using the auto-tensile tester. Each measurement was tested on least 6 specimens and the mean values were recorded. All treatments were measured three times.

#### 2.4.5. Barrier Properties

Water vapour permeability (WVP) of films was measured using a WVP tester (C390 M/H, Labthink Instruments Co., Ltd., Jinan, China). Films were cut into the same round shape (diameter: 80 mm), and tested at 38 °C and 90% RH. Oxygen permeability (OP) of films was measured using a film permeability testing machine (Classic 216, Labthink Instruments Co., Ltd., Jinan, China). Films were cut into the same circles (diameter: 100 mm) with no holes or scratches. The durations of gas replacement and test interval were 60 s and 3600 s, respectively. The WVP and OP values of films were obtained after three replicates.

#### 2.4.6. Moisture Content and Water Solubility of Film

The moisture content (*MC*) and water solubility (*WS*) measurements were obtained using the method described by Kim et al. [15] with some modifications. The films were cut into squares (side length: 5 cm), weighed (W0), and dried at 105 °C until a constant weight (W1) was achieved. After that, films were soaked in 100 mL purified water at 25 °C for 24 h. Then, films were dried at 105 °C to constant weight (W2). The *MC* and *WS* were calculated using the following equations [15]:

(2) MC(%)=(W0−W1)/W0×100%

(3) WS(%)=(W1−W2)/W1×100%

#### 2.4.7. Optical Transmittance

The UV–vis analysis was performed using the ultramicro analyzer (Nano Genius, MAPADA, Shanghai, China). The transmittance (T) spectra were registered between 190 nm and 900 nm with a scanning interval of 2 nm.

#### 2.4.8. Anti-Oxidative Slow-Release Properties

The experiment was conducted using water, 3% acetic acid aqueous solution, 10% ethanol aqueous solution, and 95% ethanol aqueous solution as simulants of water-soluble, acidic (pH < 4.5), alcoholic, and fatty foods, according to Lian et al. [16]. Briefly, films were cut and soaked in 4 kinds of food simulants, from which equal amounts of film extract were sampled at different times to measure the anti-oxidative capacity. The anti-oxidation capacity of film extract was measured with the use of total anti-oxidative capacity (T-AOC) assay kits.

### 2.5. Experiment on Application in Dried Shrimp Storage

#### 2.5.1. Preparation, Packaging, and Storage Method of Dried Shrimp

The frozen shrimps were thawed at 4 °C, then shrimps (500 g) were mixed with boiling water (1 L) and boiled for 3 min. After cooling, the shrimps were dried in constant temperature and humidity box at 60 °C, 30% relative humidity for 16 h. The dried shrimp were packaged using SFs and TFs containing 0%, 1.25%, 2.5%, 3.75%, and 5% AFE, respectively. Dried shrimps packaged in polypropylene bags were used as the control. All the packaged shrimps were stored at 37 °C for 40 days before being stored at −80 °C.

#### 2.5.2. Protein Oxidation of Dried Shrimp

Myofibrillar protein (MP) in dried shrimp was extracted using the method of Zhao et al. [17]. A protein content assay kit (biuret method) was used for measuring MP concentration. And the MP was dissolved in 20 mmol/L phosphate buffered saline (PBS) (pH 7.0) containing 0.6 mmol/L NaCl and immediately analysed. The contents of total sulphhydryl group, carbonyl group, and hydrophobicity of myofibrillars were measured as previously reported by Zhao et al. [17].

#### 2.5.3. Lipid Oxidation of Dried Shrimp

Briefly, the dried shrimp powder (100 g) was combined with a mixture of methanol and chloroform (1:2, *v*/*v*) solution (100 mL). After constant oscillating for 4 h, the solution was filtrated using a blob of absorbent cotton topped with anhydrous sodium sulphate. To obtained the shrimp lipid, the filtrate was spin steamed under reduced pressure at 40 °C for 20 min. The peroxide value (POV) of the shrimp lipid was determined according to Wang et al. [18].

The thiobarbituric acid reactive substances (TBARS) value of dried shrimp was measured according to Wang et al. [18] with some modifications. The dried shrimp powder (5 g) sample was placed in 30 mL of methanol and chloroform (1:2, *v*/*v*) solution. The mixture was homogenized at 8000 rpm for 30 s and filtered through a filter. A 5 mL aliquot of the resulting solution was mixed with 5 mL of 0.02 M thiobarbituric acid solutions. After incubation in a 90 °C water bath, the absorbance of the mixture was measured at 532 nm.

### 2.6. Statistical Analysis

Data were expressed as the mean ± standard deviation of replicate samples. Statistical analysis was carried out using SPSS Statistics 27. One-way ANOVA followed by Tukey’s test was used to assess the significant difference between the tested samples (significance of data was defined at *p* < 0.05).

## 3. Results and Discussion

### 3.1. Visual Changes

**Figure 2:**
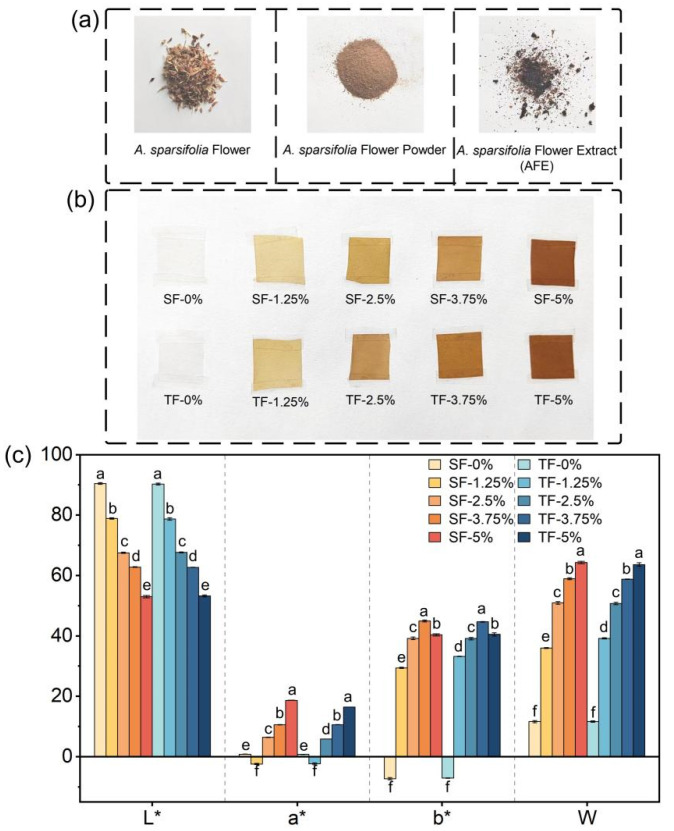
Photographs of *A. sparsifolia* flower, flower powder, and AFE (**a**), and appearance (**b**) and colour parameters (**c**) of SFs and TFs supplemented with 1.25%, 2.5%, 3.75%, and 5% AFE, respectively. Means a,b,c,d,e,f specify differences in different physical property between SF-0%, SF-1.25%, SF-2.5%, SF-3.75%, SF-5%, TF-0%, TF-1.25%, TF-2.5%, TF-3.75%, and TF-5%.

Photographs of *A. sparsifolia* flower, flower powder, and AFE are shown in Figure 2a. Figure 2b shows the appearance of SFs and TFs. In the absence of AFE, films exhibited transparency, whereas the presence of AFE at a concentration of 1.25% resulted in a yellowish-green coloration. The films exhibited a reddish-brown hue when the AFE concentration was raised to 2.5%, and the colour became darker with the escalating quantity of AFE.

The colour results are shown in Figure 2c. The films demonstrated a negative a* value at a concentration of 1.25% AFE, indicating that the chromaticity data suggested films exhibited a greenish colour when a little quantity of extract was introduced. The variations in the L* and *W* values of the films indicated that the chroma is significantly influenced by the gradient of AFE addition set in the experiment.

### 3.2. Morphology Structure

**Figure 3:**
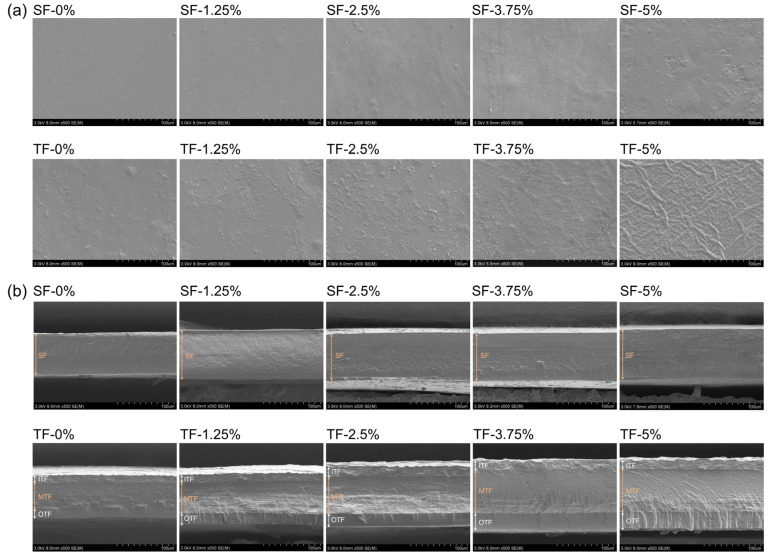
SEM micrographs (500× magnification) of the surfaces (**a**) and cross-sections (**b**) of SFs and TFs incorporated with 0, 1.25%, 2.5%, 3.75%, and 5% AFE. Abbreviations: IFT—inner layer of TF, MTF—middle layer of TF, OFT—outer layer of TF.

The surface SEM micrographs of the SFs and TFs were seen to be dense and flat (Figure 3a), indicating that the GG, PVA, and AFE were well integrated. As the addition of AFE increased, a gradual augmentation appeared in the surface roughness of both SFs and TFs. For SFs, the increase in AFE led to a corresponding augmentation in the total solid content in the film liquid. These solids were not distributed uniformly during the drying process. The surface of film finally become rough from a microscopic standpoint due to the continual evaporation of water throughout the drying process. Gan et al. [19] also reported that the introduction of curcumin into the chitosan film resulted in surface roughness. The surface microstructure of the TFs exhibited roughness as the addition of AFE increased. However, it is worth noting that the texture of TFs’ surface differed greatly from that of SFs. On one hand, this phenomenon can be attributed to the fusion and penetration caused by the GG film liquid when it was poured onto the lower surface of the TF. Additionally, the contraction resulting from water evaporation in the GG layer applied a force on the lower surface of the TF, together leading to the formation of the distinctive texture.

The cross-sectional microstructure of SFs showed a consistent and homogeneous one-layer morphology, and TFs’ cross-sectional microstructure showed a three-layer morphology which was tightly linked and had distinct boundaries (Figure 3b). In addition, the cross-sections of all films were fully intact and exhibited an absence of pores, suggesting that components present in films possess favourable compatibility [20]. Additionally, the cross-sections of films were prepared using liquid nitrogen fracture, revealing that the freezing fracture behaviour did not result in the formation of cracks in the films [21]. As shown in Figure 3b, the homogeneous and dense nature of the PVA layer contributed significantly to the effective barrier properties in TFs. The GG layer exhibits a somewhat loose structure, facilitating the slow-release properties of anti-oxidative capacity from the middle layer.

### 3.3. FTIR Analysis

**Figure 4:**
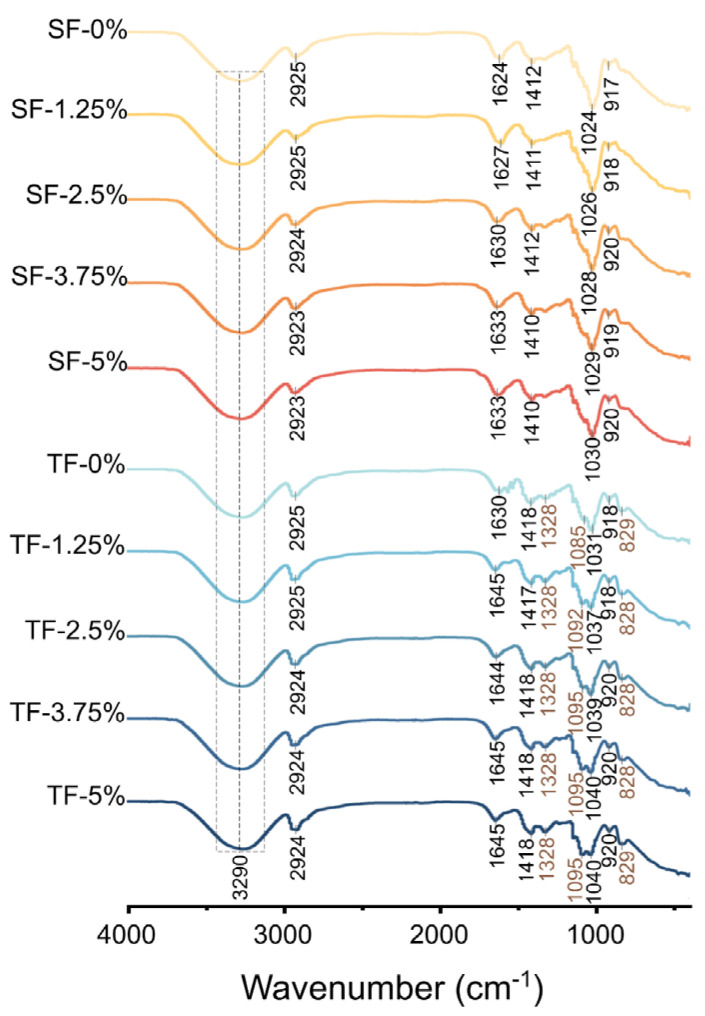
FTIR spectrum of SFs and TFs supplemented with 1.25%, 2.5%, 3.75%, and 5% AFE.

Figure 4 showed the films’ FTIR. The strong and broad absorption peak exhibited at around 3290 cm^−1^ can be attributed to the stretching vibration of O-H [22]. PVA, GG, and glycerine possessed a notable abundance of hydroxyl groups, resulting in a small variation in this peak across different film samples. The peaks at 2925 cm^−1^, 1420 cm^−1^, and 916 cm^−1^ corresponded to the stretching vibration of C-H [19], bending vibration of C-H, and swing motion of -CH_2_ [23], respectively. The positions of these peaks did not change considerably between films, owing to the existence of a substantial amount of non-reactive C-H in GG and PVA [23].

The peak at 1629 cm^−1^ could be regarded as an asymmetric stretching vibration of COO^−^ [20]. This peak exhibited a blue shift, suggesting that AFE was effectively bound to the film matrix as its addition increased [24]. The peak at 1024 cm^−1^ could be attributed to the stretching vibration of C-O. In contrast to SFs, the TFs exhibited a small blue shift at 1024 cm^−1^, suggesting the possibility of inter-layer cross-linking and the formation of new C-O [25]. The observed rise in AFE led to a small blue shift at 1024 cm^−1^, potentially indicating the formation of new C-O between the AFE and film matrix [25].

There were three additional characteristic peaks in the TFs compared to the SFs, suggesting that a new cross-linking reaction formed between the layers. The peaks at 1328 and 1091 cm^−1^ were characteristic peaks of outer PVA layer [26]. The peak at 1328 cm^−1^ corresponds to in-plane bending vibrations of C-H [27], whereas the peak at 1091 cm^−1^ arises from C-O-C symmetric stretching vibration [28]. The peak at 831 cm^−1^ was associated with the stretching vibration of the glucoside bond [29]. This may be ascribed to the presence of the pure GG layer inside the TF where an ordered network structure formed which was facilitated by the glucoside bond.

### 3.4. Optical Transmittance

**Figure 5:**
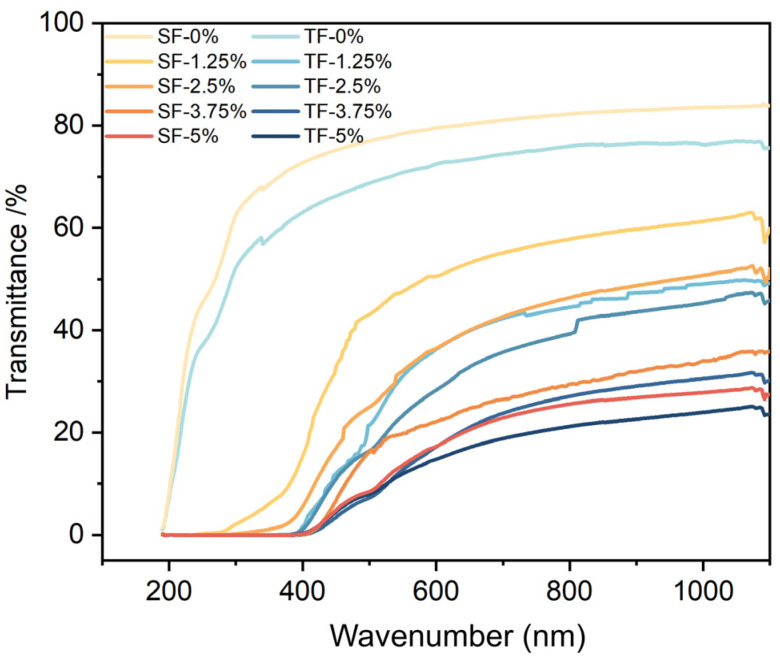
UV–vis transmittance of SFs and TFs supplemented with 1.25%, 2.5%, 3.75%, and 5% AFE.

The exposure of food to ultraviolet radiation can lead to food oxidation, causing a decrease in its nutritional content and the generation of poisonous and detrimental compounds [30]. Therefore, the efficacy of film in blocking ultraviolet radiation holds significant importance. Figure 5 showed the UV–vis transmittance of SFs and TFs, wherein different additive amounts of AFE have been included.

Figure 5 illustrates that the light transmittance of the TFs, when the same amount of AFE was applied, was comparatively lower than that of the SFs, especially in the UV range (190–400 nm). This observation suggests that the three-layer structure strengthened its light barrier properties. The research conducted by Wu et al. [27] also revealed that the double-layer film exhibited superior UV-blocking capabilities compared to the SF. Furthermore, films without AFE showed worse light barrier properties compared to films with AFE. The addition of AFE resulted in a notable decrease in light transmittance, particularly in the UV range (190–400 nm). Furthermore, as the amount of AFE increased, there was a corresponding drop in the transmittance of films. The AFE contained flavonoids, polyphenols, anthocyanins, and other aromatic compounds that possessed UV–vis absorption properties. As the concentration of AFE increased, the colour of films intensified, causing a decrease in films’ UV–vis transmittance. Prior research had also indicated that the addition of compounds into films can augment their light-blocking characteristics. The films containing bilirubin (BIL) had significant UV-blocking characteristics [30]. Wu et al. [27] discovered that films containing darker modified anthocyanin had superior UV-blocking characteristics compared to films containing lighter unmodified anthocyanin.

### 3.5. Moisture Content and Water Solubility of Films

As seen by the data presented in Table 1, there was a progressive rise in the moisture content of films as the amount of AFE added increased. The alteration in the crystalline structure of the PVA and GG blends could be attributed to the addition of AFE which contains polysaccharides, flavonoids, polyphenols, and other hydrophilic substances. Consequently, films with added AFE exhibit enhanced water-binding properties.

The water solubility is indicative of films’ ability to withstand water. Based on results showed in Table 1, it is evident that the augmentation of the AFE quantity led to the rise in the water solubility of both SFs and TFs. The reason for this phenomenon was that AFE is inherently water-soluble and, as more AFE was added to film, more film dissolved. Furthermore, it could be observed that the water solubility of the SFs was lower in comparison to the TFs which had the same quantity of AFE. This phenomenon can be attributed to the dissolution of the middle layer containing the GG and PVA blend with AFE added after the GG layer of the TFs dissolving first. The GG layer of the TFs possessed favourable water solubility, thereby leading to its dissolution in water first.

### 3.6. Barrier Properties of Films

The water vapour permeability (WVP) indicated films’ water vapour barrier properties, and the WVP values of films are presented in Table 1. The WVP value was primarily influenced by the absorption of water vapour on the surface of films, the diffusion of water through films, and the evaporation of water from the external surface [31]. The results indicated that the WVP value of the SFs was higher in comparison to the TFs. It was observed that the WVP value did not show a significant drop as the amount of AFE increased in the TFs. The enhanced water vapour barrier property of the TFs can be attributed to the fact that the efficiency of water vapour passing through three layers with different network structures was lower than that of one layer with a homogeneous network structure [32]. Simultaneously, Wang et al. [33] discovered that the water vapour barrier properties of the poly lactic acid films improved after coating it with PVA. As the amount of AFE was augmented, the WVP in the SFs exhibited a persistent rise. Small amounts of AFE did not considerably raise films’ WVP value and the WVP value significantly rose when the amount of AFE in the SFs was raised from 0% to 3.75%. This may be attributed to the interaction between AFE and the film matrix (GG and PVA), resulting in the formation of hydrogen bonds. This interaction alters the cross-linking network structure of the original film matrix (GG and PVA), leading to a decrease in the water vapour barrier properties of films [20]. However, the addition of small amounts of AFE is insufficient to significantly alter the WVP value of films (Table 1). Li et al. observed that, as the concentration of black rice extract increased, the water vapour barrier properties of films exhibited a steady decline. In a similar vein, Koosha et al. [34] discovered that the addition of higher quantities of black carrot anthocyanins in chitosan/PVC nanocomposite films resulted in a decrease in the films’ water vapour barrier properties.

The oxygen barrier properties of the packaging films play a crucial role in preserving the storage stability and overall quality of food products, which could be expressed by the value of oxygen permeability (OP) (shown in Table 1). The OP values of TFs are lower than SFs, which means TFs had better oxygen barrier properties. These enhanced properties can be attributed to the tightly interconnected network structure of the outer layer composed of PVA, which effectively impedes the transmission of oxygen [27]. Additionally, the adhesion between layers and the increased film thickness in the TFs contribute to the hindered diffusion of oxygen. Similar to the WVP, the OP value of both SFs and TFs rose as the amounts of AFE added into films increased. This could be attributed to the excessive addition of AFE being able to break down the convoluted channels within the film matrix and increase the OP, which shows that the addition of AFE reduced the oxygen resistance of films.

Similar to the WVP, the OP value of SFs exhibited an upward trend and TFs showed an insignificant change with increasing quantities of AFE incorporation. The OP’s upward trend of SFs could be related to the fact that the addition of AFE changed the convoluted channels within the film matrix [19], which meant that the addition of AFE resulted in a decrease in the films’ barrier properties to oxygen.

### 3.7. Mechanical Properties of Films

Tensile strength (TS) referred to the maximum stress exerted on films before fracture, whereas elongation at break (EAB) represented the ratio of the elongation exhibited by films at the point of fracture to their initial length (Table 1). The TS and EAB values are contingent upon the characteristics of the film matrix and the compounds added into films, and the interplay between them [35]. The TFs outperformed the SFs in terms of EAB, implying that the TFs had greater elasticity and flexibility, which might be attributable to the impact of the PVA layer [36]. Previous research has also seen a similar occurrence. Wu et al. [37] demonstrated that the addition of a polyester layer improved the TS and EAB of starch film. Similarly, Chen et al. [38] reported that the inclusion of a PVA layer boosted the EAB of a polysaccharide film. In both SFs and TFs, the TS exhibited a reduction, while the EAB shown an increase, as the AFE concentration was raised. This can be attributed to the interaction between the AFE and the film matrix, resulting in the formation of a novel cross-linked network, and the newly formed network promotes intermolecular motion in the film [39]. Akhila et al. [28] studied the characterization of PAV and guar gum film, and found the TS of the film diminished as the concentration of Ipomoea coccinea extract rose. Wu et al. [37], who tested the GG film with clitoria ternatea extract, and Goudarzi et al. [39], who researched a carrageenan–PVA electrospun fibre pad with red plum skin extract, both discovered that, as the amount of extract grew, films’ TS decreased and EAB increased.

### 3.8. Anti-Oxidative Capacity Slow-Release Properties

**Figure 6:**
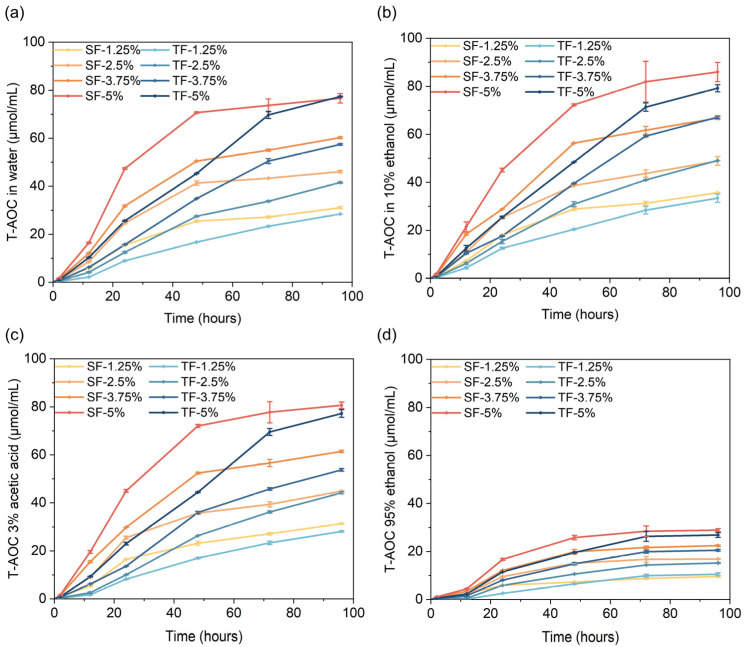
The slow release of films’ anti-oxidative capacity in 4 kinds of food simulants. The simulants are water (**a**); 3% acetic acid (**b**); 10% ethanol (**c**); and 95% ethanol (**d**). The films are SFs and TFs supplemented with 1.25%, 2.5%, 3.75%, and 5% AFE, respectively.

The pace at which anti-oxidative compounds in the packaging films were released played a crucial role in determining the longevity of the packaged food. A release rate that was too slow might result in insufficient anti-oxidative capacity in early storage, whereas a release rate that was too fast might result in insufficient anti-oxidative capacity in later storage. The release rate of anti-oxidative compounds is contingent upon their compatibility with the film matrix and the packaged food. Consequently, it is imperative to study the release rate of anti-oxidative compounds in different food systems. Figure 6 illustrates the release rate of the anti-oxidative components in films when soaked in the food simulating liquids. The result showed that the slow-release curves of the SFs and TFs exhibited different trends. The SFs showed a higher release rate of anti-oxidative capacity in the initial stage, whereas the TFs had a comparatively quicker release rate of anti-oxidative capacity in the later stages. This observation suggested that the TFs possessed a more gradual and sustained anti-oxidative capacity release impact over an extended period of time. In both SFs and TFs, there is a positive correlation between the quantity of AFE added and anti-oxidative capacity released. A comparable occurrence was documented in the investigation conducted by Gan et al. [19].

The anti-oxidative capacities released by films in different food simulating liquids at the 96th hour were as follows: 10% ethanol > 3% acetic acid > pure water > 95% ethanol. The released anti-oxidative capacity of films soaked in 95% ethanol is much lower compared to other food simulating liquids. Liu et al. [30] revealed that the compounds released from 95% ethanol at the early phase were much lower than the other food simulating liquids. However, the release efficacy of films in 95% ethanol significantly enhanced during the latter phase. The anti-oxidative capacity of films soaked in 95% ethanol did not increase to the same level as that of food simulating liquids in our study, which may be due to differences between the film matrix and anti-oxidative additives in our study and Liu et al. [30].

In summary, the TFs showed better slow-release efficacy in all food simulating liquids. All films demonstrated a slower release rate in fat simulating liquids (95% ethanol).

### 3.9. Application of Films

#### 3.9.1. Lipid Oxidation of Dried Shrimp

**Figure 7:**
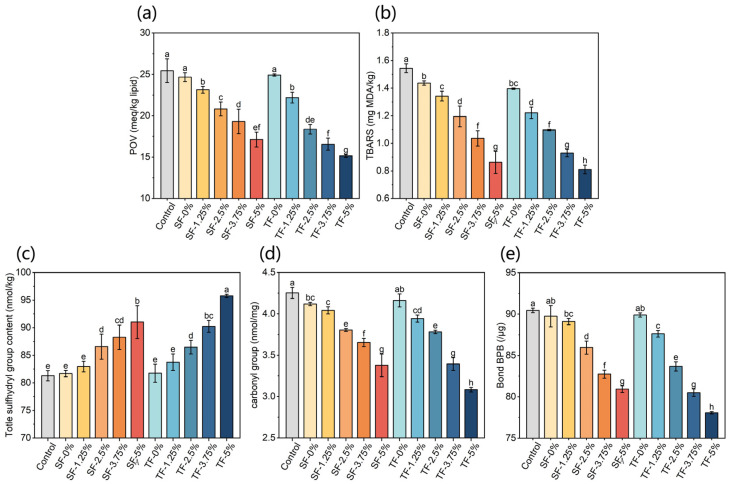
POV value (**a**) and TBARS value (**b**), and myofibrillar protein’s total sulphhydryl groups (**c**), carbonyl groups (**d**), and hydrophobicity (**e**) of dried shrimps packaged with SFs and TFs supplemented with 0%, 1.25%, 2.5%, 3.75%, and 5% AFE on the 40th day of storage. Means a,b,c,d,e,f,g,h specify differences in different physical property between SF-0%, SF-1.25%, SF-2.5%, SF-3.75%, SF-5%, TF-0%, TF-1.25%, TF-2.5%, TF-3.75%, and TF-5%.

The unsaturated fatty acids are rich in shrimp; the unsaturated fatty acids will undergo lipid oxidation to produce hydro-peroxides and malondialdehyde (MDA) during storage. The contents of hydro-peroxides and MDA in shrimp packaged in different films on the 40th day of storage were expressed by POV (Figure 7a) and TBARS (Figure 7b) values, respectively.

When the amount of AFE added into the film is 0%, there is no significant difference in the POV and TBARS value of shrimp packaged with SFs and TFs. The POV value and TBARS value of dried shrimp decreased with the increase in AFE content in films. The POV value of shrimps packaged in TFs was significantly lower than that in SFs when the AFE’s addition amount rose to 2.5–5%. And the TBARS value of shrimps packaged in TFs was significantly lower than that in SFs when the AFE’s addition amount was 1.25–3.75%. In conclusion, TF supplemented with AFE has a higher ability to slow down lipid oxidation. The TF-5% showed a reduction of 47.5% in TBARS value in relation to the control sample at the end of 40 days. Xu et al. [40] packaged fresh chicken breasts with bioactive films supplemented with 7.7% (*w*/*w*) oregano essential oil and discovered that, when chicken breasts were stored at 4 °C for 7 days, their TBARS dropped by roughly 32% compared to films without oregano essential oil. Chen et al. [41] discovered that, after 15 days of storage at 20 °C, the TBARS value of dried eel packaged with PVA films containing 2% green tea extract decreased by about 40% compared to samples packaged with films without extract.

#### 3.9.2. Protein Oxidation of Dried Shrimp

During food storage, the sulphhydryl groups of proteins are readily oxidized and form disulphide bonds within or between peptides [42]. Prolonged storage conditions can also result in the degradation of the protein’s three-dimensional conformation, the exposing of internal hydrophobic regions, and an elevation in surface hydrophobicity [43]. The presence of hydrophilic amino acids, such as lysine, arginine, asparagine, and threonine, in an oxidative environment resulted in the generation of carbonyl derivatives [44]. Hence, the levels of total sulphhydryl groups, carbonyl groups, and hydrophobicity were important indicators of protein oxidation. Figure 7 showed the total sulphhydryl content (c), carbonyl content (d), and hydrophobicity (e) of the myofibrillar protein of dried shrimp packaged in SFs and TFs after 40 days of storage. The carbonyl content and hydrophobicity of myofibrillar protein in dried shrimp exhibited a decrease after a storage period of 40 days, which was found to be directly proportional to the concentration of AFE incorporated in films. There was a significant rise in the total sulphhydryl content of dried shrimp myofibrillar protein when the amount of AFE added into the film reached 2.5%. When the amount of AFE added is below 5%, there is no statistically significant disparity observed in the overall sulphhydryl content of myofibrillar protein between dried shrimp packaged in SFs and TFs. When the amount of AFE added was set at 5%, the inhibitory impact of TFs on the oxidation of total sulphhydryl groups was shown to be significantly superior compared to that of SFs. Significantly reduced levels of carbonyl content and hydrophobicity were observed in the myofibrillar protein of dried shrimp packaged in TFs when the AFE amount added reached 2.5%, compared to SFs. The findings of the study indicate that the incorporation of AFE into films had a beneficial effect in preventing the oxidation of myofibrillar proteins in dried shrimp. Da Nobrega Santos et al. [45] employed edible film supplemented with malpighia emarginata waste extract to package beef patties and stored them under −18 °C for 60 days. Their findings demonstrated that the total protein carbonyl level of beef patties wrapped with films containing more than 4% extract was substantially lower than that of the treatment group including less than 2% extract. Furthermore, the inhibitory effect on oxidation was more pronounced with TFs compared to SFs.

### 3.10. Partial Least Squares Discrimination Analysis (PLS-DA) of Films

**Figure 8:**
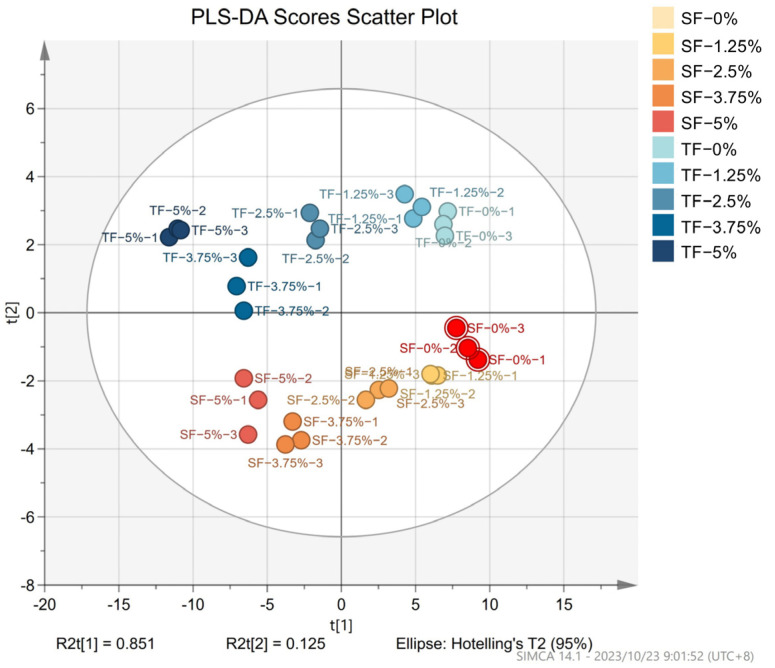
PLS-DA scores scatter plot of SFs and TFs supplemented with 0%, 1.25%, 2.5%, 3.75%, and 5% AFE.

Due to the large number of samples and analysed variables, PLS-DA was employed to gain comprehensive insights into different films [46] (shown in Figure 8). The films analysed in the PLS-DA were SFs and TFs incorporated with 0%, 1.25%, 2.5%, 3.75%, and 5% AFE. The PLS-DA was performed using films’ MC, WS, WVP, OP, TS, and EAB properties, as well as the lipid oxidation parameters (POV and TBARS) and protein oxidation parameters (total sulphhydryl content, carbonyl content, and hydrophobicity) of dried shrimps packaged with different films. In the analysis, t_[1]_ explained 0.851 of the variance and t_[2]_ explained 0.125 of the variance. Together, these two components account for more than 95% of the variance in the first two new dimensions. Figure 8 clearly demonstrates that films can be effectively separated when they have a different layer structure and AFE addition. There was a significant distance between the films containing varying quantities of AFE in the direction of the first component t_[1]_. And SFs and TFs differed significantly in the direction of the second component t_[2]_. This phenomenon demonstrated that the gradient of the amount of AFE added to films was appropriate and films’ layer structure significantly influenced their properties.

## 4. Conclusions

This study involved the development of biobased triple-layer films (TFs) with anti-oxidation properties. It was observed that the TFs exhibited superior mechanical characteristics, barrier properties, and slow-release properties in comparison to the SFs. Furthermore, the incorporation of *A. sparsifolia* flower extract (AFE) into films resulted in improved UV–vis barrier properties, moisture content, water solubility, elongation at break, and anti-oxidative capability. However, the rise in the AFE amount added was accompanied by a drop in the TS, water vapour, and oxygen barrier properties of films. The application of films in preserving dried shrimp revealed that the lipid and protein oxidation were reduced when packaged with films containing more AFE. In addition, the protective effect of the TFs was superior to single-layer films (SFs) when enough AFE (more than 2.5%) was added. Our study presented an innovative viewpoint on the development of biobased multi-layer films with anti-oxidative capacity for dried marine food preservation.

## Figures and Tables

**Table 1:** Physical properties of SFs and TFs supplemented with 1.25%, 2.5%, 3.75%, and 5% AFE.

Type	MC (%)	WS (%)	WVP (10^−15^ g m^−1^ s^−1^ pa^−1^)	OP (10^−9^ cm^3^/m^2^ d·Pa)	TS (MPa)	EAB (%)
SF-0%	19.61 ± 0.94 ^e^	32.38 ± 0.51 ^d^	1.13 ± 0.03 ^c^	2.46 ± 0.43 ^bc^	33.71 ± 1.73 ^a^	33.60 ± 3.49 ^h^
SF-1.25%	22.92 ± 1.27 ^cd^	34.71 ± 1.75 ^c^	1.14 ± 0.03 ^bc^	3.01 ± 0.32 ^ab^	30.10 ± 2.84 ^b^	43.63 ± 2.11 ^fg^
SF-2.5%	24.84 ± 0.80 ^c^	36.62 ± 0.57 ^c^	1.15 ± 0.04 ^bc^	3.26 ± 0.13 ^a^	23.95 ± 0.50 ^c^	58.43 ± 5.29 ^e^
SF-3.75%	27.76 ± 1.24 ^b^	39.22 ± 0.66 ^b^	1.18 ± 0.01 ^ab^	3.57 ± 0.43 ^a^	12.74 ± 0.44 ^d^	81.97 ± 4.45 ^c^
SF-5%	31.04 ± 2.30 ^a^	42.25 ± 0.63 ^a^	1.19 ± 0.01 ^a^	3.52 ± 0.18 ^a^	11.45 ± 0.73 ^d^	94.40 ± 2.02 ^b^
TF-0%	18.45 ± 0.70 ^e^	35.04 ± 1.12 ^c^	1.03 ± 0.01 ^ef^	2.01 ± 0.12 ^c^	35.56 ± 0.99 ^a^	37.93 ± 0.85 ^gh^
TF-1.25%	20.31 ± 1.71 ^de^	35.76 ± 1.30 ^c^	1.05 ± 0.01 ^def^	2.20 ± 0.26 ^c^	34.79 ± 2.29 ^a^	46.63 ± 0.38 ^f^
TF-2.5%	22.72 ± 1.92 ^cd^	38.98 ± 2.05 ^b^	1.03 ± 0.01 ^f^	2.21 ± 0.28 ^c^	23.28 ± 2.75 ^c^	72.03 ± 4.91 ^d^
TF-3.75%	24.57 ± 1.60 ^c^	41.00 ± 1.83 ^ab^	1.07 ± 0.01 ^de^	2.29 ± 0.01 ^bc^	12.39 ± 0.30 ^d^	91.47 ± 4.09 ^b^
TF-5%	27.40 ± 1.70 ^b^	42.85 ± 1.80 ^a^	1.07 ± 0.02 ^d^	2.29 ± 0.04 ^bc^	10.07 ± 0.41 ^d^	112.27 ± 2.28 ^a^

Abbreviations: MC—moisture content, WS—water solubility, TS—tensile strength, EAB—elongation at break, WVP—water vapour permeability, OP—oxygen permeability. Means ^a,b,c,d,e,f,g,h^ specify differences in different physical property between SF-0%, SF-1.25%, SF-2.5%, SF-3.75%, SF-5%, TF-0%, TF-1.25%, TF-2.5%, TF-3.75%, and TF-5%.

## Data Availability

The data used to support the findings of this study can be made available by the corresponding author upon request.
